# Stigma and mental health among people living with HIV across the COVID-19 pandemic: a cross-sectional study

**DOI:** 10.1186/s12879-024-09315-y

**Published:** 2024-04-22

**Authors:** Francesco Di Gennaro, Roberta Papagni, Francesco Vladimiro Segala, Carmen Pellegrino, Gianfranco Giorgio Panico, Luisa Frallonardo, Lucia Diella, Alessandra Belati, Carmen Rita Santoro, Gaetano Brindicci, Flavia Balena, Davide Fiore Bavaro, Domenico Montalbò, Giacomo Guido, Lina Calluso, Marilisa Di Tullio, Margherita Sgambati, Deborah Fiordelisi, Nicolò De Gennaro, Annalisa Saracino

**Affiliations:** 1 Clinic of Infectious Disases, Department of Precision and Regenerative Medicine and Jonian Area - (DiMePRe-J), University of Bari “Aldo Moro”, Bari, Italy; 2 Department of Translational Biomedicine and Neuroscience (DiBraiN), University of Bari ‘Aldo Moro’, Bari, Italy; 3 CamaLila Italia, Bari, Italy

**Keywords:** Mental Health, HIV, Anxiety, Depression, Post-traumatic stress disorder, Substance-related disorders, Stigma, COVID-19, Dolutegravir

## Abstract

**Background:**

Mental health (MH) is extremely relevant when referring to people living with a chronic disease, such as people living with HIV (PLWH). In fact – although life expectancy and quality have increased since the advent of antiretroviral therapy (ART) – PLWH carry a high incidence of mental disorders, and this burden has been exacerbated during the COVID-19 pandemic. In this scenario, UNAIDS has set new objectives for 2025, such as the linkage of at least 90% of PLWH to people-centered, context-specific MH services. Aim of this study was to determine the prevalence of MD in PLWH followed at the Clinic of Infectious Diseases of the University of Bari, Italy.

**Methods:**

From January 10th to September 10th, 2022, all PLWH patients accessing our outpatient clinic were offered the following standardized tools: HAM-A for anxiety, BDI-II for depression, PC-PTSD-5 for post-traumatic stress disorder, CAGE-AID for alcohol-drug abuse. Factors associated with testing positive to the four MD were explored with a multivariable logistic regression model.

**Results:**

578 out of 1110 HIV-patients agreed to receive MH screening, with 141 (24.4%) people resulting positive to at least one MH disorder. HAM-A was positive in 15.8% (*n* = 91), BDI-II in 18% (*n* = 104), PC-PTSD-5 in 5% (*n* = 29) and CAGE in 6.1% (*n* = 35). The multivariable logistic regression showed a higher probability of being diagnosed with anxiety, depression and post-traumatic stress disorder for PLWH who reported severe stigma, social isolation, psychological deterioration during the COVID-19 pandemic and for those receiving a dolutegravir (DTG)-based regimen. Moreover, history of drug use (OR 1.13; [95% CE 1.06–4.35]), family stigma (2.42 [1.65–3.94]) and social isolation (2.72 [1.55;4.84]) were found to be associated to higher risk for substance use disorder.

**Conclusions:**

In this study, stigma was a strong predictor for being diagnosed of a MH disorder among PLWH. Also, the possible role of dolutegravir as a risk factor for the onset of MH disorders should be considered in clinical practice, and MH of patients receiving DTG-containing regimens should be constantly monitored.

**Supplementary Information:**

The online version contains supplementary material available at 10.1186/s12879-024-09315-y.

## Background

Mental health (MH) is a significant global health concern as, according to the WHO definition [1], it is considered an integral part of health and a fundamental human right. Mental health is extremely relevant when referring to people living with a chronic infection, such as HIV/AIDS, even if it is not always investigated during routine follow-up visits, especially in HIV services in Low-Middle Income Countries (LMICs) [2]. While the availability of increasingly effective antiretroviral therapies (ART) has allowed for a significant reduction in disease mortality and for major increase in life expectancy of people living with HIV (PLWH) – which is now comparable to the one reported for uninfected individuals [3, 4] – it appears that PLWH taking ART are still suffering from a higher burden of comorbidities than the general population, with a long way to go [5].

Mental health challenges, including a higher prevalence of disorders such as depression, anxiety, PTSD, and substance use disorders, significantly impact the quality of life and adherence to antiretroviral therapy in PLWH [6]. In particular, among mental disorders, depression and anxiety are the most commonly seen in PLWH, but post-traumatic stress disorder (PTSD) and substance use disorders have also been found to be higher in PLWH than in the general population [7–10]. These data underline the need to delve deeper into aspects of the mental health of PLWH to find and address risk factors contributing to a higher prevalence of mental disorders among PLWH. The intersection of HIV with mental health is further complicated by stigma, which is intrinsically linked to negative mental health outcomes. Stigma not only affects self-esteem and social interactions but also has tangible consequences on the management and treatment of HIV, often leading to increased rates of depression, anxiety, and substance abuse among this population [11, 12]. Also, substance misuse presents a particularly complex issue in PLWH, harming immune recovery, increasing the burden of comorbidities and further deteriorating mental health [8, 13].

In recent years, the COVID-19 pandemic has had a significant impact on the MH of individuals around the world, as widespread social isolation, financial insecurity, increased incidence of depression and post-traumatic stress disorder [14] disproportionately affected vulnerable population such as PLWH [15]. Furthermore, several studies reported that the pandemic disrupted many aspects of HIV service delivery, with healthcare resources diverted towards COVID-19 and sudden interruptions in in-person follow-up visits and increased ART loss of adherence [16, 17]. As highlighted by the Joint United Nations Programme on HIV/AIDS (UNAIDS) [18], the COVID-19 pandemic has put at risk decades of progress in ending the AIDS epidemic. The latest report promoted the creation of new objectives for 2025, such as the adoption of people-centred approaches that result in the linkage of at least 90% of PLWH to integrated, context-specific mental health services. In line with this target, assessing the characteristics and needs of the population to which these services will be provided is of key importance.

Aim of this study was to determine the prevalence of anxiety, depression, post-traumatic stress disorder, alcohol and drug abuse and stigma among PLWH followed at the outpatient Clinic of Infectious Diseases of the University of Bari, Italy.

## Methods

### Study design and study population

A cross-sectional study was conducted from January 10th to September 10th, 2022. All PLWH patients accessing to the HIV outpatient service or hospitalized in the Infectious Diseases ward of the University Hospital Policlinico (Bari, Italy) were asked for informed consent and enrolled in this study. The only exclusion criteria was the refusal to be tested. Recruitment was conducted by convenience sampling. Ethical approval of the protocol was obtained from the Policlinico of Bari, Italy (approved n.7147 by 12.01.2022). All definitions of the mental health disorders included in the study align with Diagnostic and statistical manual of mental disorders − 5th edition (DSM-5) [19]. HIV-related stigma was defined as “the negative prejudice against PLWH who are considered socially unacceptable”, while discrimination was defined as “the hostile behavior towards PLWH that arises from stigma” [20].

### Data collection

Questionnaires were administered during routine HIV outpatient visits by either a psychologist, an infectious diseases specialist or a medical resident, who work in the HIV service and were specially trained before administering the tests to patients. Data about socio-demographic characteristics (i.e., gender, age, occupational status, and education), HIV history (years since diagnosis, AIDS events), comorbidities (diabetes, hypertension, dyslipidemia, tobacco smoke), immunological status (CD4 cell count), serologies for HBV and HCV, ongoing ART regimen, self-reported impact of COVID-19 on MH. Self-reported social and family stigma (i.e.: perception to be stigmatized by relatives) were investigated using a scale ranging from 1 to 10. All data were collected by an open-source online tool KoboToolBox [21] with questionnaires designed ad hoc for the purpose. Self-reporting biases were minimized by using validated, standardized assessment tools, ensuring anonymity and confidentiality of the responses, providing detailed clarifications, and encouraging honest responses. Once informed consent was obtained, questionnaires were administered at the end of routine outpatient follow-up visits by the attending physician. All patients enrolled in the study were offered screening for MD using the following standardized tools, that were used as outcomes of the study:

1. **Hamilton Anxiety Rating Scale (HAM-A)**: This scale consists of 14 items, with scores ranging from 0 (not present) to 4 (severe anxiety), yielding a possible total score between 0 and 56 [22].
2. **Beck Depression Inventory (BDI-II)**: The BDI-II comprises 21 items, each scored from 0 to 3, resulting in a total score of 0 to 63. Higher scores indicate more severe depressive symptoms [23].
3. **Primary Care PTSD Screen for DSM-5 (PC-PTSD-5)**: This screening tool has 5 items scored as “yes” or “no” to identify the likely presence of PTSD [24].
4. **CAGE-AID questionnaire**: Adapted to include drug use, this instrument has 4 items scored with “yes” or “no” responses to screen for alcohol and substance misuse [25].

### Statistical analysis

Data were summarized using counts and percentages for categorical variables, while medians and interquartile ranges were utilized for continuous variables. Data were stratified by mental health status, according to the positivity to one or more assessment tools. First, we explored the differences between people screening negative to all tools versus people screening positive to at least one tool. Secondly, we analysed each screening tool individually. Differences between the groups were assessed using the Mann-Whitney test for continuous measures and Fisher’s exact test for categorical data. A *p*-value of < 0.05 was used to consider differences statistically significant.

The incidence of positivity for MD screening was assessed. Factors associated with screening positive to the four MD were explored using with a multivariable logistic regression model and expressed in Odds Ratio with 95% confidence intervals within the whole study sample. Factors showing a significant association by univariate analysis were included in the multivariate analysis. Cutoff for positivity used in this study were HAM-A > 10 for anxiety [22], BDI-II > = 10 for depression [23]. PC-PTSD-5 > = 3 for post-traumatic stress disorder [24] and CAGE > = 2 for substance abuse [25].

Data were analyzed using Stata software, release 16.0. (Stata Corp., College Station, TX, USA).

## Results

Out of 1110 HIV patients acceding to our outpatient service, 578 (52%) agreed to be enrolled in the study and received the four mental health questionnaires.

Overall, 73.7% (*n* = 426) of the recruited PLWH were males, with a median age of 49 years (interquartile range [IQR] 37–56 years), and the vast majority (96.7%, *n* = 559) were of European origin. The median time to HIV diagnosis was 8 years (IQR 4–20), and almost the entire population was already on ART at the time of recruitment. The burden of comorbidity was high, since 50.8% (*n* = 294) of patients were diagnosed with either diabetes (5%), hypertension (11%), dyslipidemia (8.6%), or chronic kidney disease (3.3%), while 14% (*n* = 81) of people were co-infected with HCV, and roughly one PLWH out of ten reported substance use. Importantly, 336 (59.1%) PLWH reported to perceive moderate-severe social stigmatization, 246 (42%) people reported stigma from their family members and 227 (39.3%) reported suicidal ideation.

Overall, 141 (24.4%) people resulted positive to at least one MH disorder. HAM-A screened positive in 15.8% (*n* = 91) of the sample, BDI-II in 18% (*n* = 104), PC-PTSD-5 in 5% (*n* = 29), and CAGE-AID in 6.1% (*n* = 35). Concomitant MD were found in 14% of patients.

Descriptive statistics of all collected variables – as well as correlation to testing positive to at least one MD screening questionnaire – are shown in Table 1.

**Table 1:** Characteristics and multivariate analysis of PLWH who tested positive for at least one mental disorder vs. PLWH who tested negative for mental disorders

	Overall (*n* = 578)	Mental Disorders screening negative (*N* = *437*)	Mental Disorders screening positive (*N* = 141)	OR [95% CI]	*p*-value^1^	Adjusted *p*-value^2^
Gender, n (%)						
Male	426 (73.7)	334 (76.3)	92 (65.2%)	Ref.	Ref.	-
Female	147 (25.43)	102 (22.8%)	45 (31.9%)	1.18 [0.80;1.36]	-	0.483
Non-binary	4 (0.69%)	0 (0.93%)	4 (2.8%)	-	-	0.988
Age, median [IQR]	49 [37;56]	48.0 [36.0;56.0]	52.0 [41.0;57.0]	1.02 [0.99;1.04]	0.141	0.315
MSM (Men who have sex with men) n (%)	195 (33.74%)	100 (22.9%)	95 (67.3%)	2.94 [1.55;4.38]	0.001	0.01
Continent of origin, n (%)					0.840	
Europe	559 (96.71%)	420 (95.8%)	139 (98.4%)	Ref.	Ref.	-
Africa	12 (2.08%)	10 (2.33%)	2 (1.59%)	-	-	0.287
America	7 (1.21%)	7 (1.86%)	0 (0.00%)	-	-	0.999
Tobacco smoke, n (%)	244 (42.21%)	166 (38.1%)	78 (55.6%)	2.02 [1.14;3.60]	0.015	0.011
Years of HIV (ys of diseases), n (%)	8 [4;20]	7.00 [3.00;15.5]	11.0 [5.00;23.0]	1.03 [1.00;1.06]	0.024	0.015
Substance use, n (%)	65 (11.25%)	47 (10.7%)	18 (12.7%)	1.23 [0.49;2.81]	0.648	0.189
HBV co-infection, n (%)	25 (4.33%)	18 (4.19%)	7 (4.76%)	1.18 [0.24;4.19]	0.814	0.129
HCV co-infection, n (%)	81 (14.01%)	57 (13.0%)	24 (17.5%)	1.42 [0.64;2.99]	0.379	0.652
COVID vaccination (number of doses), n (%)						
0–1	13 (2.25%)	9 (2.33%)	4 (3.17%)	Ref.	Ref.	-
2	49 (8.48%)	33 (7.44%)	16 (11.1%)	1.06 [0.17;9.81]	0.954	0.458
3	516 (89.27%)	395 (90.2%)	121 (85.7%)	0.67 [0.13;5.31]	0.659	0.652
On ART, n (%)	576 (99.65%)	437 (100%)	139 (98.4%)	-	0.227	0.999
ART regimen, n (%)						
2 NRTI + INSTI	248 (42.91%)	170 (39.0%)	78 (55.6%)	Ref.	Ref.	-
NRTI + PI	96 (16.61%)	76 (17.4%)	20 (14.3%)	0.58 [0.24;1.30]	0.195	0.324
NRTI + INSTI	78 (13.49%)	65 (15.0%)	13 (9.52%)	0.45 [0.16;1.33]	0.091	**< 0.001**
NRTI + NNRTI	92 (15.92%)	76 (17.4%)	16 (11.1%)	0.46 [0.17;1.08]	0.075	0.809
NNRTI + INSTI	53 (9.17%)	42 (9.86%)	11 (7.94%)	0.58 [0.18;1.57]	0.295	0.138
Others	11 (1.9%)	8 (1.41%)	3 (1.59%)	0.86 [0.03;7.69]	0.903	0.918
Dolutegravir-containing regimen, n (%)	144 (24.91%)	79 (18.1%)	65 (46.0%)	3.82 [2.08;7.05]	**< 0.001**	**< 0.001**
Previous SARS CoV-2 infection, n (%)	31 (5.36%)	29 (6.51%)	2 (1.59%)	0.26 [0.01;1.35]	0.126	0.052
Comorbidities, n (%)	294 (50.87%)	213 (48.8%)	81 (57.1%)	1.39 [0.79;2.48]	0.251	0.589
Diabetes, n (%)	29 (5.02%)	15 (3.26%)	14 (9.52%)	3.12 [1.35;9.97]	**0.002**	**0.01**
Hypertension, n (%)	64 (11.07%)	39 (8.84%)	25 (17.5%)	2.19 [0.94;4.85]	0.067	0.171
CKD, n (%)	19 (3.29%)	14 (3.26%)	5 (3.17%)	1.02 [0.14;4.51]	0.977	0.148
Dyslipidemia, n (%)	50 (8.65%)	32 (7.44%)	18 (12.7%)	1.82 [0.70;4.41]	0.211	0.305
Substance use, n (%)	61 (10.55%)	43 (9.77%)	18 (15.9%)	1.75 [0.74;3.89]	0.192	0.079
AIDS events, n (%)	225 (38.93%)	156 (35.8%)	69 (48.9%)	1.44 [1.15;2.38]	**0.002**	**0.01**
HIV-related hospitalization, n (%)	161 (27.85%)	132 (30.2%)	29 (20.6%)	0.61 [0.30;1.17]	0.136	0.008
Nadir CD4 count, cell/mm3 (median, Q1, Q3)	650 [230; 800]	677 [289;800]	560 [190;695]	1.00 [1.00;1.00]	**0.012**	**0.011**
Total Pill Burden (median, Q1, Q3)	1.00 [1.00;3.00]	1.00 [1.00;3.00]	2.00 [1.00;3.00]	1.04 [0.90;1.22]	0.574	0.960
Negative to HIV-RNA at screening	559 (96.71%)	425 (97.2%)	134 (95.2%)	0.56 [0.14;2.89]	0.454	0.993
Self-reported stigma						
None (0–3)	240 (41.52%)	205 (47.0%)	35 (24.4%)	Ref.	Ref.	-
Moderate (4–7)	130 (22.49%)	82 (18.6%)	48 (34.0%)	1.98 [1.51;2.61]	**<0.001**	**0.001**
Severe (8–10)	205 (35.47%)	147 (33.5%)	58 (41.6%)	1.48 [0.88;2.12]	**0.136**	**0.004**
Family stigma	246 (42.56%)	152 (34.9%)	94 (66.7%)	2.75 [1.64;3.81]	**< 0.001**	**0.001**
Social isolation:	269 (46.54%)	173 (39.5%)	96 (68.3%)	1.68 [1.31;2.94]	**<0.001**	**0.001**
Self-perceived Impact of COVID-19 pandemic on overall mental health						
None	197 (34.08%)	169 (38.6%)	28 (20.0%)	Ref.	Ref.	-
Moderate	150 (25.95%)	116(26.5%)	34 (24.1%)	1.12 [0.49;2.48]	0.791	0.078
Severe	231 (39.97%)	152 (34.9%)	79 (55.9%)	2.43 [1.21;4.23]	**< 0.001**	**< 0.001**

ART: antiretroviral therapy; CI: Confidence intervals. CKD: chronic kidney disease; INSTI: Integrase Strand Transfer Inhibitor; NNRTI: Non-Nucleoside Reverse Transcriptase Inhibitors; NRTI: Nucleoside Reverse Transcriptase Inhibitors; OR: Odds ratio. PI: Protease Inhibitor

^1^ Mann-Whitney test or Fisher’s exact test, as appropriate

^2^ Multivariable logistic regression analysis

Ref: reference category

At multivariate analysis, differences in the distribution of the variables between PLWH with MD screening positive and MD screening negative were detected for men who have sex with men (MSM) (aOR 2.94; [95%CI 1.55–4.38]), dolutegravir-containing regimen (aOR 3.82; [2.08–7.05]), diabetes (aOR 3.12; [1.35–9.97]), AIDS-related events (aOR 1.44; [1.15;2.38]), moderate stigma perception (aOR 1.98; [1.51–2.61]), perception of family stigma (aOR 2.75; [1.64–3.81]), reported social isolation (aOR 1.68; [1.31–2.94]), and severe self-perceived impact of COVID-19 pandemic on overall MH (aOR 2.43 [1.21;4.23]). Also, factors associated with testing positive to at least one screening tool were: tobacco smoke (aOR 2.02, [1.14–3.60]), having diabetes mellitus (aOR 3.12, [1.35–9.97]), presenting AIDS events (aOR 1.44, [1.15–2.38]), receiving a dolutegravir(DTG)-based regimen (aOR 3.82, [2.08–7.05]), moderate self-reported stigma (aOR 1.98, [1.51–2.61]), social isolation (aOR 1.68, [1.31–2.94]), and self-reporting severe impact of the COVID-19 pandemic on overall MH (aOR 2.43, [1.21–4.23]).

When stratifying for MD (Table 2), self-reported severe stigma was associated with anxiety and substance use disorder (*p* < 0.05), while perceived family stigma was associated with all four screened mental health conditions. On the other hand, perceived impact of COVID-19 pandemic on mental health and DTG-based therapies were associated with anxiety disorder, post-traumatic stress disorder and depression, but not with substance use disorder.

**Table 2:** Logistic regression analysis (unadjusted and multiple adjusted estimates) for positivity to HAM-A, BDI-II, PTSD and CAGE-AID

	HAM-A positive (*n* = 91),	aOR [95%CIs]	CAGE-AID (*n* = 35)	aOR [95%CIs]	BDI-Positive (*n* = 104)	aOR [95%CIs]	PTSD (*n* = 29)	aOR [95%CIs]
Self-reported stigma, n (%)								
None (0–3)	26 (28.6)	Ref.	16 (45.7)	Ref.	44 (42)	Ref.	9 (28.6)	Ref.
Moderate (4–7)	28 (31.1)	1.76 [0.82;3.26*	10 (28.6)	1.47 [0.71;3.25]	25 (24)	1.27 [0.55;2.79]	10 (35.7)	2.76[0.68;12.1]
Severe (8–10)	37 (40.3)	1.62 [1.28;2.57]*	9 (25.7)	0.68 [0.43;1.36]*	35 (34)	1.36 [0.62;1.73]	10 (35.7)	1.36[0.34;5.88]
Family stigma, n (%)	81 (88.6)	3.44 [2.18;5.97]**	31 (88.2)	2.42 [1.65;3.94]**	62 (60)	2.46 [1.52;4.73]**	22 (78.6)	4.12 [1.89;7.10]**
Social isolation, n (%)	82 (90.9)	4.56 [2.61;7.16]*	29 (82.4)	2.72 [1.55;4.84]*	66 (64)	2.65 [1.61;3.21]**	22 (78.6	3.52 [2.09;5.03]**
Perceived impact of COVID-19 pandemic on MH, n (%)								
None (0–3)	14 (15.9)	Ref.	13 (35.3)	Ref.	27 (26)	Ref.	8 (28.6)	Ref.
Moderate (4–7)	12 (13.6)	1.25 [0.38;4.01]*	10 (28.6)	1.47 [0.71;3.25]	25 (24)	1.38 [0.58;3.29]	2 (7.14)	0.38 [0.01;2.85]
Severe (8–10)	65 (70.5)	5.23 [2.28;13.7]**	16 (47.1)	1.64 [0.91;4.00]	52 (50)	2.05 [1.37;4.30]**	19 (64.3**)**	3.13 [1.26;8.36]*
DTG-based ART(Yes), n (%)	54 (59.1)	6.52 [3.30;13.2]*	14 (41.2)	2.30 [0.79;6.34]	54 (52)	4.76 [2.49;9.19]**	15 (50.0)	2.01 [1.02;7.02]*

^1^ Mann-Whitney test or Fisher’s exact test, as appropriate

^2^ Multivariable logistic regression analysis

*p-value < 0.05

**p-value < 0.001

ART: antiretroviral therapy; DTG: Dolutegravir

On the other hand, history of drug use (aOR 1.13, [1.06–4.35]), family stigma (aOR 2.45, [1.65–3.94]) and social isolation (aOR 2.72, [1.55–4.84]) predicted diagnosis of substance use disorder (CAGE ≥2), while screening positive for post-traumatic stress disorder (PTSD-5 ≥3) was associated with family stigma (4.12, [1.89–7.10]), social isolation (aOR 3.52, [2.09–5.03]), DTG- containing regimens (aOR 2.01, [1.02–7.02]) and self-reporting severe impact of the COVID-19 pandemic on overall MH (aOR 3.13, [1.26–8.36]). The multivariable analysis for factors associated with screening positive for each study questionnaire is displayed in Table 2.

Additional Tables 1 and 2, available as supplementary material, show logistic regression analysis (unadjusted and multiple adjusted estimates) for further covariates for the positivity of BDI-II, HAM-A, CAGE-AID, and PTSD tests.

## Discussion

Among the 578 PLWH enrolled in this study, about 1 out of 4 (24%) screened positive for at least one MD, with depression (18%) and anxiety (15.8%) being the most common. According to the latest WHO World Mental Health Report [26], the global prevalence of MD was roughly 1 out of 8 people of all ages, the global prevalence of mental disorders was around 1 in 8 people of all ages, with anxiety and depression also being the most prevalent. Since then, as documented by a systematic review [27], the COVID-19 pandemic has caused a global increase in the prevalence of these mental disorders, influenced by pre-pandemic mental well-being, low-resource settings [28, 29], and socio-economic vulnerability [30, 31].

In Italy, a large study conducted on the general population both before and after the start of the COVID-19 pandemic found a variation in the prevalence of depressive symptoms closely tied to pandemic-related events such as epidemic waves and restrictions. Specifically, the prevalence rates were 6.1% in 2018–2019, 7.1% in March–April 2020, peaked at 8.2% in July–August, and then gradually decreased to levels similar to pre-pandemic times in November–December 2020 (5.9%). Notably, the study highlighted a higher risk among women and individuals experiencing financial difficulties [32]. The prevalence found in our sample was considerably higher compared to the rates reported for general Italian population during the pandemic period.

In our cohort of PLWH, a significant association was found between perceived MH deterioration during the COVID-19 pandemic and the likelihood of experiencing anxiety, depression and post-traumatic stress disorder. Other studies have also indicated an increase in support requests to mental health services among cohorts of PLWH during the pandemic [33, 34]. This trend is attributed to various factors, including limitation of access to health services, concern about the effects of SARS-CoV2 infection in immunocompromised patients, social isolation, and economic constraints [15, 33, 35]. For instance, a 2018 meta-analysis of studies involving PLWH living in the UK reported depression rates ranging from 22 to 49% [36], and anxiety rates ranging from 17 to 47%. Additionally, an observational study conducted on a large cohort of PLWH in North America found that 55.1% had at least one mental health condition, with depression (39%) and anxiety (28%) being the most prevalent [37]. These findings highlight a higher prevalence of MD among PLWH compared to the rates recorded in our study.

These significant differences in the prevalence of MD among PLWH across countries underscore the high variability influenced by factors such as healthcare system structure, social dynamics, cultural norms, economic conditions, and historical background. These differences emphasize the importance of assessing PLWH mental health status in each setting to tailor services for the early prevention, diagnosis, and management of mental disorders.

In our study, we observed an elevated risk of anxiety, depression, alcohol and drug abuse, and post-traumatic stress disorder (PTSD) among patients experiencing stigma from their social contacts and family members. As underlined by the Center for Disease Control and Prevention (CDC), social and family discrimination are mainly based on a lack of disease awareness and significantly compromise the quality of life of PLWH by leading to stigma internalization and social isolation [20]. Such environments are conducive to the development of various mental health disorders, as observed in our study. The causal pathway between stigma and these mental health outcomes can be attributed to several interlinked factors. Stigma internalization often leads to feelings of worthlessness and self-blame among PLWH, exacerbating stress psychological distress. This heightened emotional turmoil can make substance use seem appealing as a coping mechanism, further increasing the risk of developing substance use disorders [38]. Similarly, the isolation and loneliness resulting from stigma can contribute to depressive symptoms, creating a cycle of mental health challenges. Indeed, a recent report published by the International Labour Organization (ILO) found that around 40% of people globally believe that PLWH should not be allowed to work with HIV-negative people. The report also identified a correlation between positive attitudes towards PLWH and higher levels of education and disease knowledge [39]. In this context, our study confirms that stigma and discrimination have an impact on the MH of PLWH and, as reported by meta-analysis [40], show a significant association with anxiety, depression, hopelessness, loneliness, PTSD, and low self-esteem. Further studies should aim to increase our understanding of these relationships and provide evidence supporting interventions designed to increase disease awareness, reduce stigma, and offer mental health support to people living with HIV.

Moreover, in this study, a significant association emerged between DTG-based therapies and MD, in particular anxiety, depression, and post-traumatic stress disorder. The correlation between DTG and anxiety and depression is well-known, with several studies reporting anxiety and depressive manifestations that arose following the start of a DTG-containing regimen and regressed after discontinuation [41–43], even if the evidence is conflicting [44]. Regarding the association between Dolutegravir and PTSD, in contrast to our findings, a study carried out on over 500 women with HIV did not show a correlation between worsening of PTSD scores following the start or switch to therapy with Dolutegravir, while a worsening was found following a switch to therapy containing Raltegravir [45]. The mechanisms by which DTG can cause psychiatric disorders are not known, but the available studies suggest a multifactorial genesis that also includes genetic factors [46]. In line with these findings, our study supports the need to constantly monitor MH among PLWH receiving DTG-based regimens, especially in consideration of its widespread use and the increased risk of developing the MD described in this population.

We recognize several limitations: first, this is a single-centre study, and the tests were not administered to all patients followed up at our service but only to those who agreed, which may have induced a selection bias since reasons for the acceptance to undergo the tests (e.g., subjective perception of psychological discomfort) and reasons for the refusal (e.g., lack of time, denial of the MD, etc.) may have affected the outcome incidence. Second, we do not have a previous term of comparison to analyse the trend of the prevalence of MD in patients followed up at our centre and understand how much the prevalence is influenced by the COVID-19 pandemic or by other variables (e.g., DTG-based therapies). Third, recruitment was conducted by convenience sampling, without prior sample size calculation. Fourth, our data lacked the granularity required to conduct internal validation tests. Lastly, because of the small numbers, we could not distinguish the severity of a psychiatric disorder but only whether it was present or not.

## Conclusions

Our data show a high prevalence of MD among PLWH and reinforce the need for multidisciplinary health policy action with focused intervention on MH in PLWH, especially in the pandemic era. Strategies to periodically monitor the MH status of PLWH through clinical screening and specific testing are required; in this sense, it is necessary to create or implement specific integrated services that can allow early diagnosis and care for MD.

The perception of being subjected to stigma (be it familial or in an extra-familial setting) is a risk factor for the onset of MD, so it is mandatory to implement strategies, including public communication strategies, that address this issue and reduce or even eliminate stigma that is always unjustified, but even more so in the U = U (undetectable = untransmittable) era. Effective policies must be put in place to increase knowledge and awareness of HIV infection in both PLWH and the general population to combat self-stigma and discrimination.

Further studies and particular attention are required for some categories that appear to be most exposed to the risk of MD among PLWH, such as women, adolescents, and MSM. In addition, these combined and targeted efforts could improve the MH of PLWH and therefore their quality of life.

### Electronic supplementary material

Below is the link to the electronic supplementary material.

Supplementary Material 1

Supplementary Material 2

## Data Availability

The datasets used and/or analysed during the current study are available from the corresponding author on reasonable request.

## Abbreviations

ART: Anti-retroviral therapies
DTG: Dolutegravir
HIV: Human immunodeficiency virus
MD: Mental disorders
MH: Mental health
PLWH: People living with HIV
