# Supplementary material for: Stigma and mental health among people living with HIV across the COVID-19 pandemic: a cross-sectional study
Source: BMC Infect Dis. 2024 Apr 22;24:423. doi: 10.1186/s12879-024-09315-y (PMC11034033; doi:10.1186/s12879-024-09315-y)
Supplement: Supplementary file 1 — Supplementary Material 1 [file 12879_2024_9315_MOESM1_ESM.docx]

**Table 2**: Logistic regression analysis (unadjusted and multiple adjusted estimates) for positivity of following test: HAM-A and BDI

|  | **HAM-A Pos n.91 (%)** | **OR** | **p. OR** | **p. adj** | **BDI-Pos n.104 (%)** | **OR** | **p. OR** | **p. adj** |
| --- | --- | --- | --- | --- | --- | --- | --- | --- |
| **Self-reported stigma:** |  |  |  |  |  |  |  |  |
| None (0-3) | 26 (28.6) | Ref. | Ref. | - | 44 (42) | Ref. | Ref. | - |
| Moderate (4-7) | 28 (31.1) | 1.76 [0.82;3.26] | 0.187 | 0.031 | 25 (24) | 1.27 [0.55;2.79] | 0.567 | 0.088 |
| Severe (8-10) | 37 (40.3) | 1.62 [1.28;2.57] | <0.001 | 0.005 | 35 (34) | 1.36 [0.62;1.73] | 0.666 | 0.008 |
| **Family stigma** | 81 (88.6) | 3.44 [2.18;5.97] | <0.001 | <0.001 | 62 (60) | 2.46 [1.52;4.73] | <0.001 | <0.001 |
| **Social isolation** | 82 (90.9) | 4.56 [2.61;7.16] | <0.001 | <0.001 | 66 (64) | 2.65 [1.61;3.21] | <0.001 | 0.002 |
| **Suicide ideation** | 47 (52.3) | 2.32 [1.18;4.62] | 0.005 | 0.011 | 52 (50) | 2.29 [1.20;4.45] | 0.002 | 0.007 |
| **Mood related to COVID-19 pandemic:** |  |  |  |  |  |  |  |  |
| None (0-3) | 14 (15.9) | Ref. | Ref. |  | 27 (26) | Ref. | Ref. |  |
| Moderate (4-7) | 12 (13.6) | 1.25 [0.38;4.01] | 0.709 | 0.211 | 25 (24) | 1.38 [0.58;3.29] | 0.462 | 0.012 |
| Severe (8-10) | 65 (70.5) | 5.23 [2.28;13.7] | <0.001 | <0.001 | 52 (50) | 2.05 [1.37;4.30] | <0.001 | 0.006 |
| **DTG-based ART(Yes)** | 54 (59.1) | 6.52 [3.30;13.2] | <0.001 | 0.002 | 54 (52) | 4.76 [2.49;9.19] | <0.001 | 0.001 |
| **MSM** | 43 (47.2) | 1.88 [1.33;2.71] | <0.001 | 0.001 | 25 (23.8) | 0.98 [0.59;2.09] | 0.371 | 0.042 |

**Table 2 A**: Logistic regression analysis (unadjusted and multiple adjusted estimates) for positivity of following test: HAM-A and BDI, Supplementary material.

|  | **HAM-A Pos n.91 (%)** | **OR** | **p. OR** | **p. adj** | **BDI-Pos n.104 (%)** | **OR** | **p. OR** | **p. adj** |
| --- | --- | --- | --- | --- | --- | --- | --- | --- |
|  |  |  |  |  |  |  |  |  |
| Female | 30 (34.1) | - | - | 0.059 | 40 (38.0) | - | - | 0.154 |
| Age | 52.5 [43.2;57.0] | 1.02 [0.99;1.05] | 0.157 | 0.133 | 51.5 [41.8;57.0] | 1.02 [0.99;1.04] | 0.238 | 0.052 |
| Nazionality: | 28 (31.1) | 1.76 [0.82;3.26] | 0.287 | 0.071 | 25 (24.0) | 1.27 [0.55;2.79] | 0.567 | 0.088 |
| Smoker (Yes): | 37 (40.3) | 1.62 [1.28;2.57] | <0.001 | 0.005 | 35 (34.0) | 1.36 [0.62;1.73] | 0.666 | 0.008 |
| Years of HIV (ys of diseases) | 11.5 [4.00;23.0] | 1.03 [1.00;1.06] | 0.082 | 0.126 | 12.0 [5.00;25.8] | 1.04 [1.01;1.07] | 0.009 | 0.018 |
| Drugs use (Yes): | 81 (88.6) | . 4.56 [2.61;7.16] | <0.001 | 0.001 | 62 (60.0) | . 2.86 [1.61;3.21] | <0.001 | 0.002 |
| SARS COV-2 vaccination (n. of doses) |  |  |  |  |  |  |  |  |
| 0-1 | 2 (2.27) | Ref. | Ref. | - | 5 (4.00) | Ref. | Ref. | - |
| 2 | 6(6.82) | 0.84 [0.08;27.5] | 0.901 | 0.043 | 10 (10.0) | 0.69 [0.10;6.59] | 0.718 | 0.131 |
| 3 | 83 (90.9) | 1.04 [0.16;27.4] | 0.975 | 0.034 | 89 (86.0) | 0.50 [0.10;4.03] | 0.465 | 0.334 |
| Therapy: |  |  |  |  |  |  |  |  |
| 2 NRTI + INSTI | 54 (59.1) | Ref. | Ref. | - | 64 (62.0) | Ref. | Ref. | - |
| NRTI + PI | 8 (9.09) | 0.85 [0.16;1.03] | 0.231 | 0.1298 | 14 (14.0) | 0.51 [0.19;1.22] | 0.134 | 0.078 |
| NRTI + INSTI | 10 (11.4) | 0.32 [0.26;1.41] | 0.910 | 0.038 | 6 (6.00) | 0.25 [0.46;1.37] | 0.014 | 0.001 |
| NRTI + NNRTI | 12 (13.6) | 0.88 [0.16;1.23] | 0.212 | 0.077 | 8 (8.00) | 0.29 [0.08;1.10] | 0.015 | 0.136 |
| NNRTI + INSTI | 7 (6.82) | 0.67 [0.16;1.23] | 0.888 | 0.178 | 8 (8.00) | 0.53 [0.14;1.53] | 0.252 | 0.029 |
| Others | 0 (0.00) | - | - | 0.999 | 4 (2.00) | 1.02 [0.03;9.11] | 0.990 | 0.168 |
| Comorbidity | 54 (59.1) | 1.49 [0.78;2.91] | 0.232 | 0.211 | 62 (60.0) | 1.57 [0.85;2.98] | 0.152 | 0722 |
| Drug use: | 10 (11.4) | 1.05 [0.33;2.72] | 0.928 | 0.286 | 17 (16.0) | 1.71 [0.67;3.98] | 0.247 | 0.909 |
| AIDS | 29 (31.8) | 0.75 [0.37;1.48] | 0.412 | 0.024 | 52 (50.0) | 1.88 [1.21;3.51] | 0.047 | 0.741 |
| NadirCd4 | 560 [208;753] | 1.00 [1.00;1.00] | 0.416 | 0.022 | 426 [172;753] | 1.00 [1.00;1.00] | 0.015 | 0.172 |
| Pill Burden HIV | 1.00 [1.00;1.00] | 0.70 [0.29;1.67] | 0.420 | 0.152 | 1.00 [1.00;1.00] | 0.37 [0.12;1.18] | 0.092 | 0.029 |
| Total Pill Burden | 2.00 [1.00;3.00] | 1.04 [0.87;1.24] | 0.657 | 0.103 | 2.00 [1.00;3.00] | 1.08 [0.92;1.27] | 0.322 | 0.345 |
| Last CD4 | 766 [664;1003] | 1.00 [1.00;1.00] | 0.885 | 0.296 | 752 [667;988] | 1.00 [1.00;1.00] | 0.867 | 0.437 |
| HIV.RNA<20 cp | 85 (93.2) | 0.35 [0.09;1.83] | 0.194 | 0.995 | 100 (96.0) | 0.72 [0.16;5.49] | 0.711 | 0.999 |
| Hospitalization related to HIV: | 20 (22.7) | 0.73 [0.32;1.51] | 0.402 | 0.469 | 23 (22.0) | 0.68 [0.32;1.38] | 0.299 | 0.004 |
