# Supplementary material for: Stigma and mental health among people living with HIV across the COVID-19 pandemic: a cross-sectional study
Source: BMC Infect Dis. 2024 Apr 22;24:423. doi: 10.1186/s12879-024-09315-y (PMC11034033; doi:10.1186/s12879-024-09315-y)
Supplement: Supplementary file 2 — Supplementary Material 2 [file 12879_2024_9315_MOESM2_ESM.docx]

**Table 3**:Logistic regression analysis (unadjusted and multiple adjusted estimates) for positivity of following test: CAGE-AID and PTSD

|  | **CAGE-AID Pos n. 35 (%)** | **OR** | **p. OR** | **p. adj** | **PTSD Pos**  **n = 29 (%)** | **OR** | **p. OR** | **p. adj** |
| --- | --- | --- | --- | --- | --- | --- | --- | --- |
| Self-reported stigma: |  |  |  |  |  |  |  |  |
| None (0-3) | 16 (45.7) | Ref. | Ref. | **-** | 9 (28.6) | Ref. | Ref. |  |
| Moderate (4-7) | 10 (28.6) | 1.47 [0.71;3.25] | 0.902 | 0.860 | 10 (35.7) | 2.76[0.68;12.1] | 0.153 | 0.282 |
| Severe (8-10) | 9 (25.7) | 0.68 [0.43;1.36] | **0.028** | **0.025** | 10 (35.7) | 1.36[0.34;5.88] | 0.661 | 0.374 |
| Family stigma | 31 (88.2) | 2.42 [1.65;3.94] | **0.004** | **0.001** | 22 (78.6) | 4.12 [1.89;7.10] | **0.002** | **0.001** |
| Social isolation | 29 (82.4) | 2.72 [1.55;4.84] | **<0.001** | 0.002 | 22 (78.6) | 3.52 [2.09;5.03] | **0.001** | **0.001** |
| Suicide ideation | 15 (41.2) | 1.38 [0.65;2.01] | 0.043 | 0.297 | 17 (57.1) | 1.88 [1.65;3.55] | 0.005 | 0.002 |
| Mood related to COVID-19 pandemic: |  |  |  |  |  |  |  |  |
| None (0-3) | 13 (35.3) | Ref. | Ref. | - | 8 (28.6) | Ref. | Ref. | - |
| Moderate (4-7) | 10 (28.6) | 1.47 [0.71;3.25] | 0.902 | 0.860 | 2 (7.14) | 0.38 [0.01;2.85] | 0.381 | 0.151 |
| Severe (8-10) | 16 (47.1) | 1.64 [0.91;4.00] | 0.700 | 0.237 | 19 (64.3) | 3.13 [1.26;8.36] | **<0.001** | 0.026 |
| DTG-based ART | 14 (41.2) | 2.30 [0.79;6.34] | 0.122 | 0.188 | 15 (50.0) | 2.01 [1.02;7.02] | 0.038 | 0.007 |

**Table 3B**: Logistic regression analysis (unadjusted and multiple adjusted estimates) for positivity of following test: CAGE-AID and PTSD Supplementary material

|  | **CAGE-AID Pos n.35 (%)** | **OR** | **p. OR** | **p. adj** | **PTSD Pos n.29 (%)** | **OR** | **p. OR** | **p. adj** |
| --- | --- | --- | --- | --- | --- | --- | --- | --- |
| Female | 4 (11.8) | - | - | **0.033** | 9 (28.6) | - | - | 0.210 |
| Age | 49.0 [44.0;56.0] | 1.00 [0.96;1.04] | 0.955 | 0.958 | 52.5 [35.0;57.0] | 1.01[0.96;1.06] | 0.724 | 0.678 |
| Smoker (Yes): | 19 (52.9) | 1.59 [0.58;4.42] | 0.364 | 0.369 | 14 (50.0%) | 1.40[0.46;4.28] | 0.549 | 0.115 |
| Years of HIV (ys of diseases) | 10.0 [5.00;21.0] | 1.02 [0.98;1.07] | 0.343 | 0.069 | 13.5 [3.50;22.8] | 1.03[0.98;1.08] | 0.206 | 0.237 |
| HBV Co-infection: | 0 (0.00) | - | . | 0.997 | 0 (0.00) | - | . | 0.996 |
| HCV Co-infection: | 4(11.8) | 0.86 [0.12;3.25] | 0.844 | 0.073 | 3 (14.3) | 1.08[0.15;4.26] | 0.921 | 0.110 |
| SARS COV-2 vaccination (n. of doses) |  |  | 0.601 |  |  |  |  |  |
| 0-1 | 0 (0.00) | Ref. | Ref. |  | 2 (7.14) | Ref. | Ref. |  |
| 2 | 0 (0.00) | - | . | 0.999 | 2 (7.14) | 0.29[0.01;12.4] | 0.467 | 0.603 |
| 3 | 35 (100) | - | . | 0.998 | 25 (85.7) | 0.28[0.04;7.63] | 0.353 | 0.115 |
| 2 NRTI + INSTI | 18 (52.9) | Ref. | Ref. |  | 15 (50.0) | Ref. | Ref. |  |
| NRTI + PI | 7(17.6) | - | . | 0.296 | 2 (7.14) | . - | . | 0.404 |
| NRTI + INSTI | 2 (5.88) | - | . | 0.059 | 2 (7.14) | . - | . | 0.104 |
| NRTI + NNRTI | 4 (11.8) | - | . | 0.740 | 6 (21.4) | . - | . | 0.109 |
| NNRTI + INSTI | 4 (11.8) | - | - | 0.357 | 4 (14.3) | . - | - | 0.094 |
| Others | 0 (0.00) | - | - | 0.998 | 0 (0.00) | . - | - | 0.998 |
| Comorbidity | 15 (41.2) | 0.67 [0.23;1.82] | 0.431 | 0.170 | 12 (42.9) | 0.72 [0.23;2.17] | 0.561 | 0.148 |
| Drug use | 4 (11.8) | 1.13 [1.06;4.35] | 0.879 | 0.821 | 2 (7.14) | 0.68 [0.03;3.63] | 0.706 | 0.403 |
| AIDS | 12 (35.3) | 0.92 [0.30;2.53] | 0.871 | 0.732 | 9 (28.6) | 0.67 [0.17;2.10] | 0.508 | **0.009** |
| NadirCd4 | 560 [100;650] | 1.03 [1.00;1.08] | 0.116 | **0.001** | 605 [290;775] | 1.00 [1.00;1.00] | 0.758 | **0.027** |
| Pill Burden HIV | 1.00 [1.00;1.00] | 0.17 [0.01;2.42] | 0.190 | 0.232 | 1.00 [1.00;1.00] | 0.61 [0.12;3.15] | 0.553 | 0.291 |
| Pill Burden | 1.00 [1.00;1.00] | 0.93 [0.67;1.27] | 0.634 | 0.256 | 1.50 [1.00;2.00] | 1.06 [0.80;1.39] | 0.706 | 0.219 |
| Last CD 4 | 700 [650;861] | 1.00 [1.00;1.00] | 0.358 | 0.782 | 695 [650;704] | 1.00 [1.00;1.00] | 0.198 | 0.355 |
| HIV RNA<20 cp (Yes): | 31 (88.2) | 0.20 [0.04;1.58] | 0.111 | 0.974 | 29 (100) | . 2.20 [0.84;3.58] | . 0.634 | 0.998 |
| Hospitalization related to HIV: | 4 (11.8) | 0.35 [0.05;1.28] | 0.123 | 0.218 | 7 (21.4) | 0.71 [0.15;2.40] | 0.608 | 0.911 |
